# Two stage treatment of a proximal humeral fracture-dislocation with vascular injury: Case report of a multidisciplinary approach

**DOI:** 10.1016/j.tcr.2021.100547

**Published:** 2021-10-25

**Authors:** Lorenzo Maria Di Giacomo, Fabrizio Marzano, Andrea Zaganelli, Valerio Pace, Rosario Petruccelli, Giuseppe Rinonapoli, Auro Caraffa

**Affiliations:** Trauma & Orthopaedics Department, “S.M. della Misericordia Hospital”, University of Perugia, Piazzale Gambuli 1, 06100 Perugia, Italy

**Keywords:** Proximal humeral fracture, Fracture-dislocation, Vascular injury, Brachial plexus injury, Multidisciplinary approach

## Abstract

Proximal humeral fracture-dislocation associated with neurovascular injury is rare events, associated with poorer outcomes and higher risk of complications. A multidisciplinary approach including the orthopaedic and vascular department is essential in treating such kind of injury. The goal of the treatment is to restore the vascular supply and stabilize the fracture. Usually the orthopaedic surgical stabilization provides a stable substrate for the vascular repair.

We report a case of 70 years old woman who sustained a 4 part proximal humerus fracture-dislocation with vascular injury at the level of the transition of the subclavian into axillary artery. Because of the impending severe limb ischemia, the priority of the treatment was given to vascular surgical intervention with a by-pass procedure. After 14 days a reverse shoulder prosthesis was thought to be the best alternative in the second stage surgery. At 18 months follow-up we achieved good clinical and radiological outcomes. Although a lack of consensus on the priority of treatments, we achieved good result following our proposed algorithm of treatment.

## Introduction

Proximal humeral fractures are among the most common injuries in the elderly population. In complex injury patterns they could be associated with dislocation [1], [2].

Since the proximity of the proximal humerus to neurovascular structures, nerve and/or vascular lesions are cause of concern and may occur [2].

However only few cases of proximal humeral fracture-dislocation have been reported to be associated to neurovascular injuries [3], [4]. A very small number of brachial plexus neuropathy cases are reported, with complete recovery within few months. Vascular injuries associated with such pattern of fracture are even rarely reported and described in the literature. However these types of injury are shown to be associated with worse prognosis and more invasive and complex treatments [3], [4].

We report a case of a proximal humerus fracture-dislocation associated with subclavian-axillary artery lesion, treated multidisciplinary with a two-stage procedure by the vascular and Trauma & Orthopedics (T&O) team. Priority was attributed to the vascular repair (in view of the impending limb ischemia) and it was followed by a second stage operation with the execution of a reverse shoulder arthroplasty procedure.

## Case report

A 70 years old woman presented to our local Accident & Emergency (A&E) Department after a low energy fall in a domestic environment at night time. Past medical history included only hypertension.

**Fig. 1:**
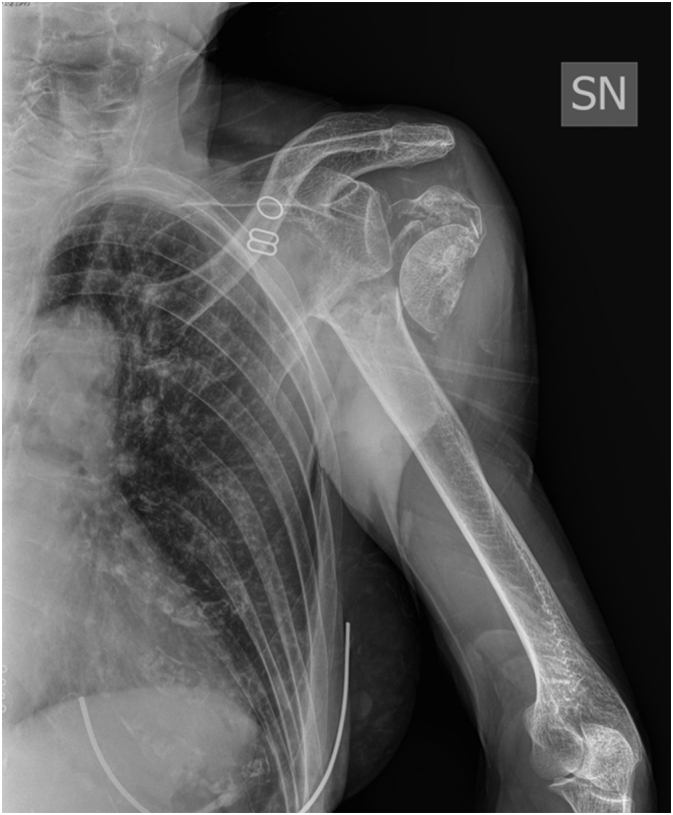
Plain X-ray at the admission.

The patient was provided with the routine triage and A&E evaluation. Plain radiographs were performed at presentation, showing a left 4 parts proximal humeral fracture with an anterior subcoracoid dislocation of the humeral head (Fig. 1). Cervical and Chest X-Rays were negative for fracture. Therefore the patient was referred to our Trauma & Orthopaedic Department for the management of the injury and was admitted under our care. However it must be highlighted that the patient was referred to our Department with a delay compared to the recommended standard management algorithm. We thought this was due to a mixture of multifactorial issues (transports, personnel shortage, night time, initial evaluation and clinical stabilization).

Clinical examination revealed intense localized pain, with no signs of active bleeding nor presence of significant bruising or haematomas. The range of motion (ROM) of the shoulder was obviously very limited. An abnormal shoulder profile was also noted. The distal neurology (both sensitive and motor) was found intact on examination. The hand was warm and pink with a capillary refill less than 2 s. However the radial pulse was absent at this point.

Vital parameters, neurocognitive status, respiratory and abdominal examination were all normal.

Her initial Hemoglobin was 12,4 g/dl and the other laboratory results were within the normal range.

**Fig. 2:**
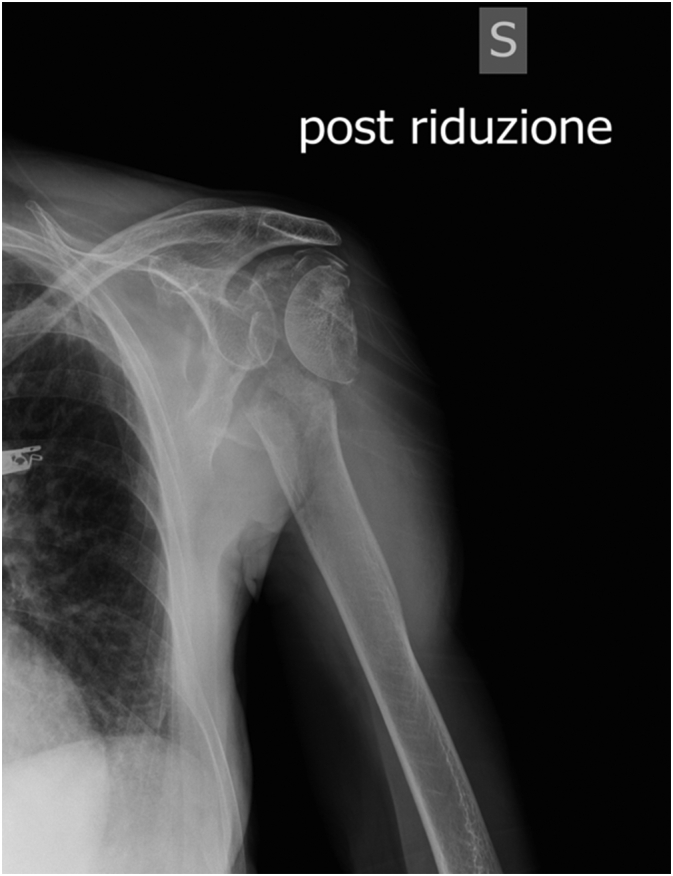
X-ray after attempt to reduction.

Given this scenario, it was decided to attempt close reduction straight after admission. The procedure was performed under sedation and anaesthetist assistance. However complete joint congruency was not achieved (Fig. 2). As the distal pulse was still absent, the patient was referred and urgently assessed by the vascular team. The clinical evaluation, an Ecocolordoppler was performed showing an interruption of the vascular supply of the radial and ulnar artery.

**Fig. 3:**
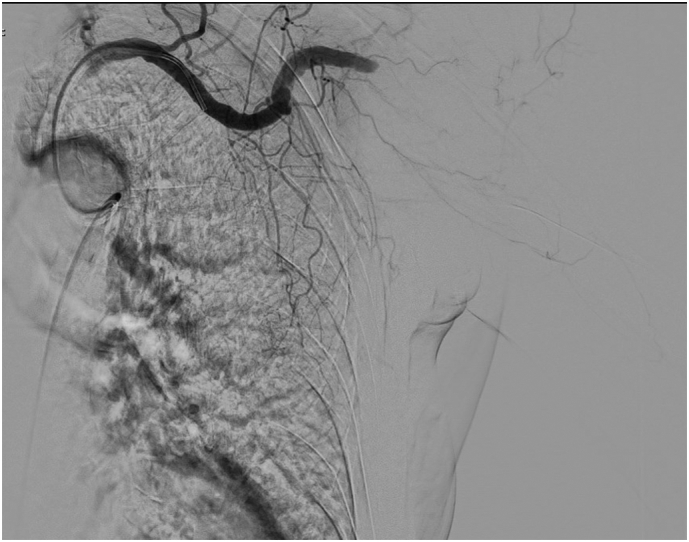
Angiography showing occlusion between subclavian and axillary artery.

At this point the patient was taken urgently to the Radiology Department for an angiography. This revealed a total occlusion of the subclavian arterial circulation in proximity of the junction between subclavian and axillary artery (Fig. 3) After these results the procedure was converted from endovascular to open vascular surgery, with the aim to reinstitute adequate blood flow and save the upper limb. A medial graft from the saphenous vein was taken at the level of the proximal third of the thigh; a 20 cm graft was obtained. A subclavicular and sovracubital approach was performed and the harvested saphenous graft bypassed in correspondence of the two incisions. At the end of the procedure good revascularization was radiologically confirmed and the distal pulse reappeared. Hb dropped to 9,9 g/dl during the first post-op hours and no transfusion was necessary.

On day 2 post-op the patient was discharged with the left arm in a sling. In order to prevent the bypass failure and with multidisciplinary consensus, the orthopaedic surgical intervention had to be postponed by 2 weeks.

Since the fracture pattern, the age and the risk for avascular necrosis, a reverse shoulder prosthesis was found to be indicated to treat this case. It was performed under general anesthesia, in beach chair position, and with an old fashioned deltopectoral approach. The cephalic vein protected and lateralized, the head found posteriorly dislocated and 180° rotated, the head removed, the tuberosities identified and tagged. After glenoid reaming, the small R Metal-Back (Lima Corporate, Villanova San Donato del Friuli, Italy) and a 36 mm eccentric glenosphere with 2 screws implanted. The humeral canal was progressively prepared, and a size 18 press-fit finned stem with metaphyseal engagement system implanted.

The stability of the implant and ROMs were tested under total muscular relaxation of the patient. A polyethylene standard reverse liner was chosen. The tuberosities were reinserted, the wound closed in layers.

A post-op X-ray showed good position of the components without signs of loosening. A sling was worn 24/7 for two weeks and encouragement to actively move from day 1 post-op the ipsilateral elbow, wrist and fingers was given. Passive mobilization of the shoulder was started at 2 post-op weeks, whilst active mobilization in elevation, extrarotation and abduction was allowed from the 4th week. A follow up X-ray was scheduled at 1, 3 and 6 months, together with clinical evaluation (provided also 12 and 18 months post-op).

**Fig. 4:**
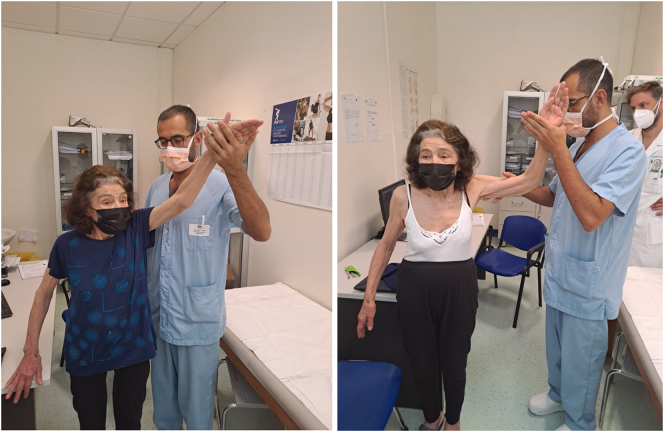
Range of motion at 18 follow-up.

The patient revealed a quite significant level of joint stiffness in the first three post-op months, but exhibited improvements over the next months. In fact the Quick Dash score is 22.0 point and ROM 110° in active elevation and 100° in active abduction 18 months after surgery, so that the patient could achieve satisfactory execution of the daily activities (Fig. 4).

**Fig. 5:**
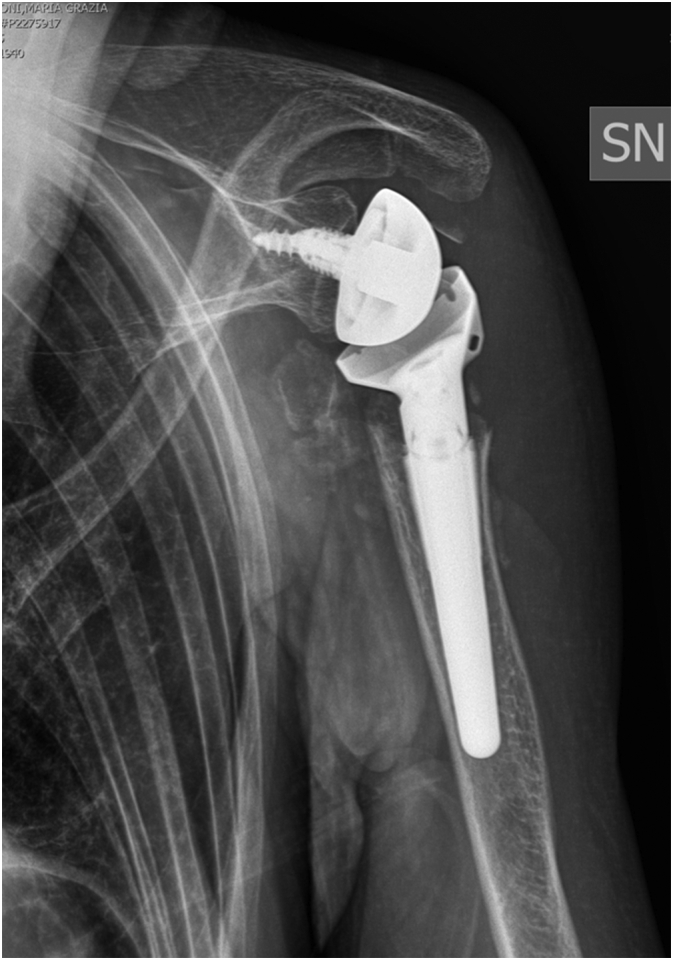
Postoperative X Ray of the shoulder at 6 month follow-up.

The X-ray at last follow up showed good position of prosthesis and no signs for instability, loosening or infection at 6 month follow-up (Fig. 5).

The patient was also followed up by the vascular team and the upper arm exhibited good vascularization in the early post-op period, and no sequelae were identified.

## Discussion

Proximal humeral fracture-dislocations are rare events, even more if associated with vascular injury with an incidence of 6.4 per 10,000 humeral fracture [3], [4]. In the literature there are only few case reports on this association of injuries.

Upper limb vascular injury occur because of penetrating and cutting injuries and only very few cases because of humeral fracture [3], [4]. Usually the mechanism of injury is associated with direct trauma [5], maneuvers of reduction in most cases or with surgical humeral head removal during the arthroplasty procedure. This complication occurs predominantly in the distal third of the axillary artery in terms of rupture, dissection of intimal layer or thrombosis [6], [7]. In our case, the direct trauma associated with the medialization of humeral diaphysis led likely to the axillary injury, since his tethered position between anterior and posterior circumflex vessels and scapular vessels, that may make the artery more susceptible [5].

Our patient exhibited a transluminal complete arterial rupture in the region between the subclavian and axillary artery, just before the axillary artery gives off his branches for the shoulder girdle. Although a rare complication, suspicion of arterial injury when dealing with this type of fractures is mandatory, It is thought that predisposition to artery lesions in such injuries is given by the presence of artheriosclerosis, open fractures, neuropathies and in male gender [4].

Unfortunately (as in our case) it is not always possible to detect an arterial vessel injury at presentation as it often presents subtly, commonly because of the presence of collateral circulation able to mask a more evident presentation of the vascular injury. The distal pulse could be preserved in some cases and mimic a non-severe condition. Drapanas et al. reported a palpable pulse in 27% of such patients despite the type of injury sustained [8].

Therefore it is crucial to properly and fully assess the patient on admission (including an accurate distal neurovascular assessment), monitoring the vital parameters and check the routine blood results. If doubts arise, or in case of high index of suspicion for vascular or neurological impairment, a multidisciplinary approach including the A&E doctors, Radiologists and Vascular and Orthopaedic surgeons and the execution of appropriate radiological investigations (Ecodoppler, angiography) are advocated. This could allow early identification of complications and early appropriate treatments, which could involve limb salvage procedures.

We believe that this type of multidisciplinary approach is paramount to reduce the risk of significant complications and sequelae. Moreover a missed or delayed diagnosis of more than 6–8 h might lead to dramatic consequences such as upper limb ischemia and amputation [3]. It has been shown that the risk of impending limb ischemia decreases by 4 h in more proximal arterial lesions [3], [5], [9].

Regarding the treatment's chronology, an initial orthopaedic treatment of the injury (reduction and internal fixation or prosthesis implantation) in patient with unstable patterns of fractures is commonly recommended, to provide a stable substrate for the following vascular repair. This is thought to prevent secondary vascular repair failure by anatomical instability or secondary dislocation [3], [4], [5], [6], [7], [8], [9]. However the severe vascular injury with impending limb ischemia and the slight delay for his diagnosis has brought the multidisciplinary team to decide toward prioritization of the vascular surgical intervention in order to prevent limb ischemia and sequelae, and postpone by 2 weeks the orthopaedic surgical intervention in order to avoid jeopardizing the outcome of the vascular bypass, which was deemed as absolute priority in this specific scenario. In case of impending limb ischemia, the majority of the Authors have suggested a prioritized vascular surgical intervention against orthopaedic surgical interventions [5].

In our opinion and given the very good final outcome both in terms of vascularization and function of the shoulder, the multidisciplinary team took the best possible choice and achieved the best possible results in this particular situation.

An under sedation fracture reduction attempt is mandatory in the presented fracture-dislocation pattern (especially in presence of an associated vascular injury) in order to minimize the vascular trauma and his consequences.

The described pattern of fracture could be treated with open reduction and internal fixation procedures, external spanning fixation or with an arthroplasty procedure. Two weeks delayed surgery is thought to be associated with more challenging procedures, higher complications' rates and poorer functional and clinical outcomes. The choice of performing a RSA was related to the injury's pattern (degree of fragmentation and displacement and presence of vascular injury, with likely impairment of the blood supply to the proximal part of the humerus), age and overall risk of avascular necrosis. Good outcomes were achieved even given the delay of 2 weeks from presentation.

Axillary neuropathy is also often associated to this type of injuries [10]. A fundamental intrinsic prerequisite for a successful RSA is proper function of the deltoid muscle. Therefore a full and accurate neurological examination of the axillary nerve must be performed, although not always completely feasible because of the potentially scares compliance of the patient (e.g. pain, discomfort). It is reported that 20–70% of such cases could present a neuropathy affecting the function of the limb. This is deemed to be due to the direct traumatic insult, or to the presence of a haematoma (and his reorganization) or post-fracture oedema [3], [4].

Currently there is no established treatment algorithm for the presented type of injury. Therefore a sufficiently big cohort study feels to be necessary in order to create a universally accepted management algorithm.

We believe that all the aforementioned key aspects and milestones for an appropriate management of such injuries were taken into account by the multidisciplinary team, with optimal coordination and performance of the most adequate interventions. This is testified by the good restoration of shoulder function and good return to daily activities without any significant impairment, showed at the 18 months post-op follow up.

## Conclusions

A significant vascular injury associated with a proximal humeral multifragmentary fracture-dislocation is a severe and complex injury pattern, carrying high risks of mechanical and vascular failure. We believe that a multidisciplinary approach including the radiology, orthopaedic and vascular departments is essential in such cases, as showed by previous literature, together with prompt and full clinical examination, including accurate distal neurovascular findings and their monitoring. However a lack of consensus on the priority of the vascular or orthopaedic treatment still exists. If diagnosis is delayed and a severe limb ischemia is impending, the priority should be given to the vascular surgical intervention and a reverse shoulder arthroplasty (RSA) is a good alternative in the second stage surgery. We achieved good final results following this pathway.

## Funding

No funding was needed.

## Declaration of competing interest

All authors disclose any financial and personal relationships with other people or organizations that could inappropriately influence (bias) their work. Examples of potential conflicts of interest include employment, consultancies, stock ownership, honoraria, paid expert testimony, patent applications/registrations, and grants or other funding.
